# Effects of CYP2C19 variants on the metabolism of tapentadol *in vitro*

**DOI:** 10.22038/IJBMS.2022.56996.12710

**Published:** 2022-05

**Authors:** Ren-ai Xu, Ping Fang, Zhize Ye, Mingming Han, Jian-Ping Cai, Guo-Xin Hu

**Affiliations:** 1 The First Affiliated Hospital of Wenzhou Medical University, Wenzhou, Zhejiang, China; 2 The First Affiliated Hospital, Zhejiang University School of Medicine, Hangzhou, Zhejiang, China; 3 School of Pharmaceutical Sciences, Wenzhou Medical University, Wenzhou, Zhejiang, China; 4 The Key Laboratory of Geriatrics, Beijing Institute of Geriatrics, Institute of Geriatric Medicine, Chinese Academy of Medical Sciences, Beijing; 5 Hospital/National Center of Gerontology of National Health Commission, Beijing, China

**Keywords:** Cytochrome P450, CYP2C19 variants, Drug metabolism, Genetic polymorphism, Tapentadol

## Abstract

**Objective(s):**

This study aims to evaluate the catalytic activities of 31 CYP2C19 alleles and their effects on the metabolism of tapentadol *in vitro*.

**Materials and Methods:**

Insect microsomes expressing the CYP2C19 alleles were incubated with 50–1250 μM tapentadol for 40 min at 37 °C and terminated by cooling to -80 °C, immediately. Tapentadol and N-desmethyl tapentadol were analyzed by a UPLC-MS/MS system. The kinetic parameters Km, Vmax, and intrinsic clearance (Vmax/Km) of N-desmethyl tapentadol were determined.

**Results:**

As a result, the intrinsic clearance (V_max_/K_m_) values of most variants were significantly altered, while CYP2C19.3 and 35FS had no detectable enzyme activity. Only one variant, N277K, showed no significant difference from CYP2C19.1B. Two variants CYP2C19.29 and L16F displayed markedly increased intrinsic clearance values of 302.22% and 199.97%, respectively; whereas 24 variants exhibited significantly decreased relative clearance ranging from 0.32% to 79.15% of CYP2C19.1B. Especially, CYP2C19.2G, 2H, R124Q, and R261W exhibited a drastic decrease in clearance (>80%) compared with wild-type CYP2C19.1B.

**Conclusion:**

As the first study of all aforementioned alleles for tapentadol metabolism, the comprehensive data *in vitro* may provide novel insights into the allele-specific and substrate-specific activity of CYP2C19.

## Introduction

The cytochrome P450 superfamily is one of the most important drug-metabolizing enzymes in the liver. It metabolizes more than 90% of current therapeutic drugs that undergo phase I metabolism (1, 2). CYP2C19, a major member of the cytochrome P450 mixed-function oxidase system, plays a critical role in the metabolism of xenobiotics in the body and is responsible for the metabolism and elimination of approximately 10% of daily used clinical drugs (3). In recent years, numerous important drugs have been identified as major CYP2C19 substrates, including antiepileptic drugs (S-mephenytoin), proton pump inhibitors (omeprazole and lansoprazole), antiplatelet drugs (clopidogrel), antidepressants (imipramine and citalopram), anti-HIV drugs (nelfinavir), and sedative hypnotics (diazepam) (4-6). The human CYP2C19 gene exhibits significant genetic polymorphisms between individuals. Different alleles could contribute to various pharmacokinetics and pharmacodynamics, which may cause undesirable adverse effects or therapeutic failures, such as prolonged sedation and unconsciousness after administration of diazepam at standard dosages in poor metabolizers (PMs) (7). 

To date, more than 30 alleles of CYP2C19 have been identified in the Human CYP Allele Nomenclature Committee website (http://www.cypalleles.ki.se/cyp2c19.htm). In a recent study, Hu *et al.* found 24 novel variants after screening all nine exons of CYP2C19 in 2127 unrelated healthy Chinese subjects (8). Among them, CYP2C19 *2E, *2F, *2G, *2H, *2J, *3C, *29, *30, *31, *32, and *33 have been named as new alleles by the Human CYP Allele Nomenclature Committee. In a later study, 24 newly reported novel CYP2C19 isoforms were highly expressed to assess the enzymatic activity of these variants on two commonly used probe substrates omeprazole and S-mephenytoin *in vitro* (9). 

Tapentadol is the latest centrally acting analgesic and has broad efficacy for more diverse types of pain with reduced opioid-related side effects and superior tolerability to classical opioids, such as morphine (10-14). In November 2008, tapentadol was approved by the United States Food and Drug Administration as an immediate release (IR) prescription drug for the management of moderate-to-severe acute pain followed by approval of the extended-release (ER) tablets for chronic pain treatment in August 2011 (12, 15). The metabolism of tapentadol is mainly via glucuronidation (Phase II metabolism) by UGT1A9 and UGT2B7 enzymes to tapentadol-O-glucuronide (16, 17). In parallel, tapentadol also undergoes phase I oxidative reactions via CYP2C19 to its main metabolite N-desmethyl tapentadol (12, 16, 18). Thus, the exploration of CYP2C19 gene polymorphisms on tapentadol metabolism can be meaningful. In our study, besides wild-type CYP2C19*1A and CYP2C19*1B, we systematically analyzed the enzymatic characteristics of 29 CYP2C19 alleles (5 alleles were previously reported, and 24 novel alleles with nonsynonymous coding changes) toward tapentadol *in vitro*.

## Materials and Methods

***Chemicals and materials***

Tapentadol (purity 98%) and its main metabolite N-desmethyl tapentadol (1.0 mg/ml in methanol) were obtained from Shanghai Canspec Scientific Instruments Co., Ltd (Perfemiker, Shanghai, China), and Carbamazepine (internal standard, IS) was purchased from Sigma-Aldrich Company (St. Louis, Mo, USA). The reduced nicotinamide adenine dinucleotide phosphate (NADPH) regenerating system was obtained from Promega (Madison, WI, USA). P450 cytochrome b5 microsomes and recombinant human CYP2C19 expressed in the microsomes from Spodoptera frugiperda 21 (Sf21) insect cells were gifted from Beijing Hospital (Beijing, China). Ultrapure water was freshly purified by a Milli-Q A10 System (Millipore, Billerica, MA, USA). High-performance liquid chromatography (HPLC) grade organic solvents and liquid chromatography-mass spectrometry (LC-MS) grade acetonitrile were purchased from Merck (Darmstadt, Germany), LC-MS grade formic acid (FA, 98% purity) was purchased from J&K Scientific Ltd. (Shanghai, China), and all other reagents used were of analytical grade. 

***Instrumentation***

Samples were analyzed by ultra-performance liquid chromatography-tandem mass spectrometry (UPLC-MS/MS) using a Waters ACQUITY H-Class and a Waters XEVO TQS triple-quadrupole mass spectrometry (MS) (Waters Corp.) with an electrospray ionization source. Masslynx 4.1 software (Waters Corp.) was used to control the instrument and process the data of the samples. 

***Conditions for enzymatic activity analysis***

According to the previously reported method, Zhou* et al.* constructed the dual-expression baculovirus vectors p-FastBac-OR-CYP2C19 and highly expressed CYP2C19 variants in the microsomes of Sf21 insect cells (19). The carbon monoxide different spectra method was used to detect the concentration of each of the recombinant CYP2C19 holoproteins in microsomal proteins. In our study, the incubation mixture as the final system consisted of microsomes 5 pmol CYP2C19.1 or other CYP2C19 mutants, purified cytochrome b5, tapentadol, and 100 mmol/L potassium phosphate buffer (pH 7.4). Tapentadol was initially prepared in methanol solution and the concentrations of tapentadol used in the incubation mixture for kinetic analysis were 50, 250, 500, 750, 1000, and 1250 μM. The total concentration of methanol in the mixture was less than 0.5%. The reaction was allowed to preincubate for 5 min in a Fisher shaking water bath. Then NADPH regenerating system was added to start the reaction at 37 °C in a final volume of 200 μl; and incubations proceeded at 37 °C for 40 min. Reactions were terminated by cooling to -80 °C immediately. Then 400 μl acetonitrile and 50 μl carbamazepine (IS, 400 ng/ml) were added to the incubation mixture. After vortexing for 2 min and centrifuging at 13000 rpm for 10 min, the supernatant was 1:9 diluted with water, and 2 μl of the mixture was injected into the UPLC-MS/MS system for analysis. A six-point standard curve was used to quantify N-desmethyl tapentadol. Incubations were performed in triplicate and data was presented as the mean ± SD; and the incubation conditions have been optimized to ensure that incubation time and concentration in the metabolic reaction are within the linear range.

***Chromatographic conditions***

The liquid chromatographic separation was carried out using an ACQUITY UPLC-MS/MS and performed on a Waters ACQUITY UPLC BEH C18 column (2.1 mm × 50 mm, 1.7 µm), with an inline 0.2 mm stainless steel frit filter connected to it. The column temperature was maintained at 40 °C, while the samples in the auto-sampler room were kept at 4 °C. The initial mobile phase consisted of solvent A (water containing 0.1% formic acid) and solvent B (acetonitrile) at a flow rate of 0.4 ml/min by injection volume of 2 μl. A gradient elution program was employed as follows: 0–0.5 min (70%–70%A), 0.5–1.0 min (70%–10% A), 1.0–1.8 min (10%A), and 1.8–2.3 min (10%–70%A). The total run time was 3 min. Under the above appropriate conditions, the retention times of N-desmethyl tapentadol, tapentadol, and carbamazepine were 0.51, 0.53, and 1.5 min, respectively. 

***Mass spectrometric conditions***

A Waters XEVO TQS triple-quadrupole MS was set to positive electrospray ionization in multiple reaction monitoring mode. The multiple reaction monitoring (MRM) transitions were *m/z* 222.04 → 107.16, *m/z* 208.12 → 106.98, and *m/z* 237.1 → 194.2 for tapentadol, N-desmethyl tapentadol, and carbamazepine, respectively. The collision energy for tapentadol, N-desmethyl tapentadol and carbamazepine was set at 25 V, 20 V and 20 V, respectively; and the cone voltage was set at 40 V for tapentadol, 35 V for N-desmethyl tapentadol, and 40 V for carbamazepine.

***Statistical analysis***

Michaelis-Menten curves and enzyme kinetic parameters (K_m_ and V_max_) were performed by nonlinear regression curve fitting using GraphPad Prism 5 (GraphPad Software Inc., SanDiego, CA, USA). Statistical analysis was carried out with the Statistical Package for the Social Sciences (version 17.0; SPSS Inc., Chicago, IL, USA) by one-way analysis of variance (ANOVA) together with the Dunnett’s test to analyze differences in catalytic activity between CYP2C19.1 and other CYP2C19 mutants. (*P*<0.05 represents statistical significance).

## Results

**Figure 1:**
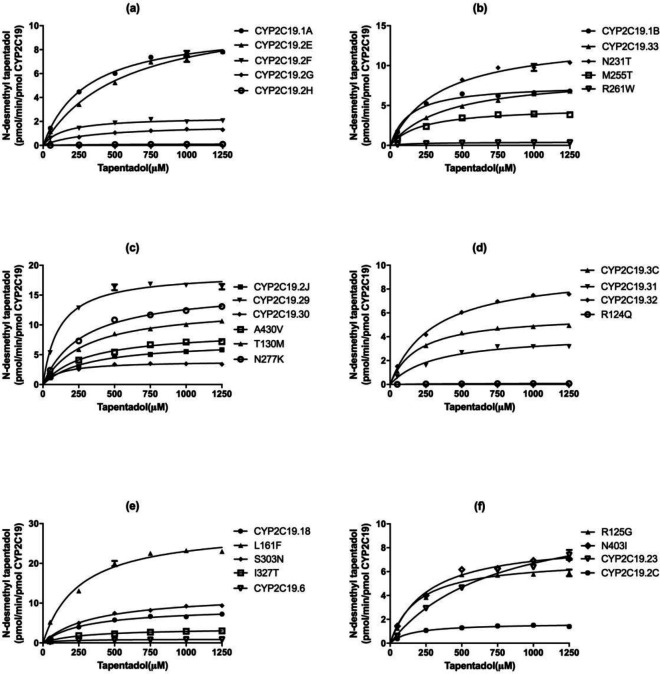
Michaelis-Menten curves of the enzymatic activity of the wild-types and 29 variants toward tapentadol N-demethylation (each point represents the mean±S.D. of three parallel experiments)

In our study, the catalytic activities of the wild-types CYP2C19.1A, CYP2C19.1B and 29 CYP2C19 variants (CYP2C19.2E, .2F, .2G, .2H, .2J, .3, .3C, .29, .30, .31, .32, .33, .23, .6, 2C, .18, 35FS, R124Q, M255T, R261W, I327T, T130M, A430V, N231T, S303N, R125G, N403I, L16F, and N277K) were detected using tapentadol as substrate. Intrinsic clearance (C_lint_) was determined as the ratio of V_max_/K_m_, which was used as evaluation criteria for each variant of CYP2C19 *in vitro* metabolic activity on tapentadol. The Michaelis-Menten curves plots for each of the CYP2C19 variants are shown in Figure 1; the corresponding kinetic parameters of N-desmethyl tapentadol estimated for wild-types and 29 CYP2C19 variants are summarized in Table 1.

As shown in Table 1, when compared with the wild-type CYP2C19*1B, except occasionally a few allelic variants, most CYP2C19 variants displayed considerable differences in K_m_ or V_max_ values, thereby possibly altering the intrinsic clearance (V_max_/K_m_) values of variants. Definitely, according to the intrinsic clearance values when compared with CYP2C19.1B, 29 CYP2C19 variants could be classified into categories: CYP2C19.3 and 35FS were too weak to produce N-desmethyl tapentadol, resulting in no detectable enzymatic activity; and according to the statistical analyses, only one variant N277K was without significant difference (101.67% relative clearance) from CYP2C19.1B; two variants (CYP2C19.29 and L16F) exhibited a distinctly decreased V_max_ value and/or a much lower K_m_ value with wild-type, resulting in higher intrinsic clearance (302.22% and 199.97% relative clearance, respectively); the remaining 24 variants (CYP2C19.2E, .2F, .2G, .2H, .2J, .3C, .30, .31, .32, .33, .23, .6, .2C, .18, R124Q, R125G, T130M, N231T, M255T, R261W, S303N, I327T, N403I, and A430V) exhibited reduced intrinsic clearance in different degrees (0.32%–79.15% relative clearance).

## Discussion

CYP2C19 is a highly polymorphic enzyme and metabolizes numerous therapeutic drugs. The study of CYP2C19 gene polymorphism, which gives rise to important interindividual and interethnic variation in the metabolism of several agents and may result in adverse effects and therapeutic failures, can provide references to the clinical research. According to research, CYP2C19 is involved in the metabolism of tapentadol to its main metabolite, N-desmethyl tapentadol (20). Furthermore, it was reported that tapentadol was associated with significant toxic clinical effects such as respiratory depression and coma, and severe outcomes consistent with an opioid agonist (21). Therefore, it is necessary to evaluate the effects of 31 CYP2C19 variants on the metabolism of tapentadol *in vitro*. 

To better understand the effects of CYP2C19 variants on the metabolism of tapentadol *in vitro*, we analyzed the 29 CYP2C19 variants in detail. Although the wild-type CYP2C19*1A was used as the control group in our previous studies (9, 22), there is increasing evidence indicating that the wild-type allele CYP2C19*1B, having 991A>G (I331V), is rather common in the Asian population, especially with high frequency (>90%) in Chinese and Japanese. Thus, in our study, the enzymatic activities of CYP2C19*1B were used as the control group (23-25).

As we all know, the most prevalent alleles CYP2C19*2 (681G>A, splicing defect) and CYP2C19*3 (636G>A, Trp212Stop) have been extensively studied in different populations and they account for >99% of PM alleles in the oriental population (26-29). CYP2C19*2 has been reported to have the highest frequency in Asian populations and accounts for approximately 75–85% of the defective alleles in both white and Japanese PMs (30). In our work, CYP2C19.2E, .2F, .2G, .2H, and .2J had a significant decrease in enzyme activities towards tapentadol compared with CYP2C19.1A and CYP2C19.1B. Especially, CYP2C19.2G and CYP2C19.2H exhibited a drastic decrease in clearance (<10%) owing to marked decreases in V_max_ and huge increases in K_m_. These results were consistent with the previous work and also confirmed that CYP2C19*2 could be classified as a PM allele for tapentadol, which indicates that it is appropriate to use our CYP expression and *in vitro* metabolism system for analyzing the catalytic activities of the other CYP2C19 variants. The CYP2C19*3 allele, which consists of a single base pair 636G>A mutation on exon 4, creates a premature stop codon. As a consequence, the kinetic parameters of CYP2C19.3 could not be determined and it exhibited no catalytic activity on tapentadol (31). As the 35FS variant also generates null functions and could not be detected because of insertion of five nucleotides, CCTAC, on position 101 of exon 1, which causes a frameshift of amino acid 35 in protein translation (25).

Aside from the well-known poor metabolic variants CYP2C19.2 and CYP2C19.3, most of the variants exhibited much lower metabolic activities than CYP2C19.1B in our study. For example, it is worth mentioning that R124Q exhibited the lowest intrinsic clearance value (<1%). Three other variants R261W, CYP2C19.6, and CYP2C19.2C, also displayed more than 80% decrease in intrinsic clearance of tapentadol relative to the wild-types. As for these results, Dai *et al*. (2015) speculated that the lower expression level of the protein may be one of the main reasons for the reduction in enzymatic activity (9). Patients carrying the CYP2C19*2G, *2H, *6, *2C, R124Q, and* R261W *alleles should pay more attention to the dose of CYP2C19 substrates, especially, which have a narrow therapeutic window.

In addition, we also found that some of the CYP2C19 variants showed a similar trend in the enzyme activity, but others were not in accordance with the results of previous research (9, 22). For example, CYP2C19.29 exhibited obviously decreased catalytic activity toward methadone, S-mephenytoin, and omeprazole (9, 22), whereas in our study CYP2C19.29 substantially increased V_max_ values and decreased K_m_ values, which showed it significantly increased relative clearance (521.36% of CYP2C19.1A). These differences from previous studies indicated that CYP2C19 had substrate-specific alterations in metabolic activity and we speculate that it is owing to the different affinity of enzyme and substrate.

**Table 1:** Kinetic parameters for N-demethylation activities of wild-types and 29 mutant CYP2C19 alleles against tapentadol

Variants	V_max_ (pmol/min/pmol P450)	K_m_ (μM)	C_lint_(V_max_/K_m_)	Relative clearance (% of CYP2C19.1A)	Relative clearance (% of CYP2C19.1B)
CYP2C19.1A	9.92±0.37	301.73±12.59	0.0329±0.0002	100.00	/
CYP2C19.1B	7.65±0.29	135.77±15.74	0.0567±0.0047	/	100
CYP2C19.2E	11.39±0.07*^, #^	553.67±1.99*^, #^	0.0206±0.0001*^, #^	62.70*	36.34^#^
CYP2C19.2F	2.37±0.09*^, #^	148.93±21.20*	0.0161±0.0018*^, #^	48.93*	28.37^#^
CYP2C19.2G	1.75±0.08*^, #^	344.33±48.95^ #^	0.0051±0.0005*^, #^	15.51*	8.99^#^
CYP2C19.2H	0.15±0.01*^, #^	630.17±79.59*^, #^	0.0002±0.0000*^, #^	0.74*	0.43^#^
CYP2C19.2J	7.26±0.15*	310.27±14.52^ #^	0.0234±0.0006*^, #^	71.29*	41.32^#^
CYP2C19.3	ND	ND	ND	ND	ND
CYP2C19.3C	5.84±0.10*^, #^	195.60±6.85*^, #^	0.0299±0.0006*^, #^	91.00*	52.75^#^
CYP2C19.29	18.64±1.03*^, #^	109.55±13.48*	0.1711±0.0117*^, #^	521.36*	302.22^#^
CYP2C19.30	3.92±0.10*^, #^	119.50±8.66*	0.0329±0.0015^#^	100.20	58.09^#^
CYP2C19.31	4.19±0.07*^, #^	319.60±36.17^ #^	0.0132±0.0012*^, #^	40.05*	23.22^#^
CYP2C19.32	9.58±0.05^ #^	299.93±9.59^ #^	0.0320±0.0010^ #^	97.40	56.46^#^
CYP2C19.33	8.89±0.22*	396.20±37.16*^, #^	0.0225±0.0016*^, #^	68.67*	39.81^#^
35FS	ND	ND	ND	ND	ND^#^
L16F	28.93±0.16*^, #^	256.17±18.39*^, #^	0.1133±0.0073*^, #^	344.98*	199.97^#^
R124Q	0.11±0.03*^, #^	636.77±233.15*^, #^	0.0002±0.0000*^, #^	0.56*	0.32^#^
R125G	7.08±0.48*	190.23±32.87*	0.0376±0.0037*^, #^	114.37*	66.30^#^
T130M	13.15±0.79*^, #^	295.27±46.71^ #^	0.0450±0.0044*^, #^	136.55*	79.15^#^
N231T	13.50±0.78*^, #^	346.83±43.01^#^	0.0391±0.0027*^, #^	119.00*	68.98^#^
M255T	4.78±0.02*^, #^	222.77±8.09*^, #^	0.0215±0.0007*^, #^	65.29*	37.85^#^
R261W	0.42±0.03*^, #^	190.07±36.25*	0.0023±0.0003*^, #^	6.86*	3.98^#^
N277K	16.08±0.14*^, #^	279.33±6.46^ #^	0.0576±0.0013*	175.39*	101.67
S303N	12.34±0.16*^, #^	362.43±25.35*^, #^	0.0341±0.0019^ #^	103.84	60.19^#^
I327T	3.71±0.04*^, #^	287.20±29.35^#^	0.0130±0.0012*^, #^	39.64*	22.98^#^
N403I	8.64±0.19*	257.10±17.88*^, #^	0.0337±0.0016^#^	102.53	59.43^#^
A430V	9.08±0.29*	300.37±26.17^ #^	0.0303±0.0017*^, #^	92.27*	53.49^#^
CYP2C19.23	11.82±1.46^ #^	762.73±147.71*^, #^	0.0157±0.0013*^, #^	47.60*	27.59^#^
CYP2C19.6	0.95±0.05*^, #^	138.37±32.49*	0.0071±0.0015*^, #^	21.17*	12.27^#^
CYP2C19.2C	1.67±0.01*^, #^	148.27±3.36*	0.0113±0.0003*^, #^	34.35*	19.91^#^
CYP2C19.18	8.93±0.22*	296.87±14.29^#^	0.0301±0.0007*^, #^	91.68*	53.14^#^

## Conclusion

In summary, we systematically assessed the enzymatic activity of 31 CYP2C19 allelic isoforms on the metabolism of tapentadol *in vitro*. Thus far, this is the first report of all these alleles with respect to tapentadol metabolism. Our results showed that most variants exhibited significantly decreased enzymatic activities towards tapentadol N-demethylation. Therefore, we speculate that patients who carry these defective alleles should reduce drug dosage in order to avoid adverse reactions, and, of course, further clinical studies are required to confirm our inference. Moreover, these data complement the database of enzymatic activity of CYP2C19 variants and may provide an informative reference for further clinical studies regarding individual variation in tapentadol efficacy and toxicity.

## Authors’ Contributions

XRA, FP Study conception and design, Perform experiment; YZZ Data analyzing and draft manuscript preparation; HMM Critical revision of the paper; CJP, H GX Supervision of the research, and Funding Acquisition.

## Conflicts of Interest

The authors state no conflicts of interest.
